# The maximum 2D diameter column of the notch as the most important bone risk indicator compared with the posterior tibial slopes for ACL injury based on computed tomography: Analysis using machine learning approach

**DOI:** 10.1002/jeo2.70630

**Published:** 2026-01-11

**Authors:** Ke Xiao, Song Wu, Benjamin Rothrauff, Bosomtwe Kwabena Richmond, Damaris Topola Boko, Jiewen Luo, Chi Liang, Yangbo Cao, Yunheng Yao, Jinshen He

**Affiliations:** ^1^ Department of Orthopaedic Surgery Third Xiangya Hospital of Central South University Changsha Hunan China; ^2^ Atrium Health Musculoskeletal Institute Charlotte North Carolina USA

**Keywords:** ACL injury, machine learning, radiomic, shapley additive explanation

## Abstract

**Purpose:**

To develop a machine learning model capable of predicting the bony risk of non‐contact anterior cruciate ligament injury, thereby enabling the identification of factors that contribute to such injuries.

**Methods:**

Data were collected from 400 cases of non‐contact ACL‐injured and 200 ACL‐intact control subjects using Computed Tomography between March 2022 and June 2025. Thirteen features, encompassing demographic, clinical, and radiomic data, as well as six different algorithms, were utilised to develop predictive machine learning models. Shapley Additive Explanations (SHAP) analysis was subsequently performed on the optimal model.

**Results:**

The Maximum 2D Diameter Column values for the non‐contact ACL injury group and the intact ACL group were 31.12 ± 2.92 mm (95% confidence interval [CI]: 30.83–31.40, *p* < 0.05) and 32.37 ± 3.07 mm (95% CI: 31.94–32.80, *p* < 0.05). The Extreme Gradient Boosting classifier was identified as the optimal predictive model, achieving an area under the precision–recall curve of 0.94, the highest among all models evaluated. SHAP analysis revealed that the most predictive feature was the Maximum 2D Diameter Column of the notch, defined as the largest pairwise Euclidean distance between tumour surface mesh vertices in the row‐slice plane, followed by the lateral and medial posterior tibial slope.

**Conclusion:**

The machine learning model developed in this study demonstrated excellent predictive performance for non‐contact ACL injuries. The Maximum 2D Diameter Column was the most important predictor, followed by the lateral and medial posterior tibial slope.

**Level of Evidence:**

Level III.

**Abbreviations**
ACL: anterior cruciate ligament
AUROC: area under the receiver operating characteristic curve
CI: confidence interval
DICOM: Digital Imaging and Communications in Medicine
LGBM: light gradient boosting machine
LR: logistic regression
MD: mean difference
MLP: multilayer perceptron
NWI: notch width index
PACS: Picture Archiving and Communication System
PTS: posterior tibial slope
RF: random forest
ROI: region of interest
SVM: support vector machine
XGB: extreme gradient boosting classifier

## INTRODUCTION

The anterior cruciate ligament (ACL) is a critical structure responsible for maintaining anterior‐posterior and rotational stability of the knee joint. ACL injuries are prevalent and costly sports‐related injuries that can significantly impair the quality of life for affected individuals. The annual incidence rate is estimated to be 1 in 3000 [10, 23].

Non‐contact ACL injuries arise from various risk factors, such as anatomical variations, neuromechanical factors, biomechanical movements, genetic predispositions, and hormonal influences [6, 25, 29, 30]. Among them, anatomical variations have received the most extensive research attention. Numerous studies suggest that parameters such as the posterior tibial slop of the medial and lateral compartments, the intercondylar fossa index, the femoral α angle, femoral intercondylar width, femoral condyle width, and the morphology of the intercondylar fossa all influence the risk of ACL injury [3, 4, 7, 16, 17]. However, these studies have not clearly identified which specific anatomical feature exerts the most significant effect on the likelihood of ACL injury.

Radiomics is a field that involves extracting a large number of quantitative features from medical images to uncover underlying disease characteristics and biological information [14]. Radiomics has been used to guide the automatic classification of meniscus injury and ACL injury based on artificial intelligence [22, 31]. This approach enables the identification of latent patterns embedded within medical imaging data. Current research on the morphology of the intercondylar notch remains primarily focused on its clinical and anatomical features. Therefore, investigating the radiomic features of the intercondylar notch to predict ACL injuries is warranted.

Machine learning, a subfield of artificial intelligence, enables the direct modelling of target tasks based on training data [18]. Unlike traditional statistical methods, which typically assume a linear relationship between predictive features and outcomes, machine learning algorithms, such as tree‐based models and support vector machines with non‐linear kernels, can capture complex, non‐linear relationships between predictors and outcome [26]. The objective of this study is to analyse bony risk factors and develop an associated osseous risk prediction model using machine learning techniques to identify individuals at high risk of trauma and enable targeted interventions for this population. Thus, the hypotheses of this study were as follows: (1) the machine learning algorithm would predict non‐contact ACL injuries with high predictive performance; (2) there would be important attributing factors in predicting non‐contact ACL injuries according to the maximum 2D diameter column of the notch based on radiomics.

## PATIENTS AND METHODS

The study includes three main components: data screen, machine learning algorithm, and visualisation. Institutional Review Board approval was obtained before performing the study (Approval No. 2025‐S471). Cases meeting the inclusion criteria were retrieved from the Picture Archiving and Communication System (PACS) for the period from March 2022 to June 2025. The exclusion criteria were as follows: patients with (1) history of knee fracture; (2) Kellgren–Lawrence grade 3–4 osteoarthritis; (3) rheumatoid arthritis; and (4) history of notchplasty. To analyse osseous risk factors for ACL injury, this retrospective study included 600 cases, which were divided into two groups: patients with non‐contact ACL injuries and control subjects with intact ACL. All ACL injuries were confirmed by intraoperative arthroscopy. The dataset was constructed by measuring the participants' demographic data, clinical parameters, and radiomic features.

**Figure 1:**
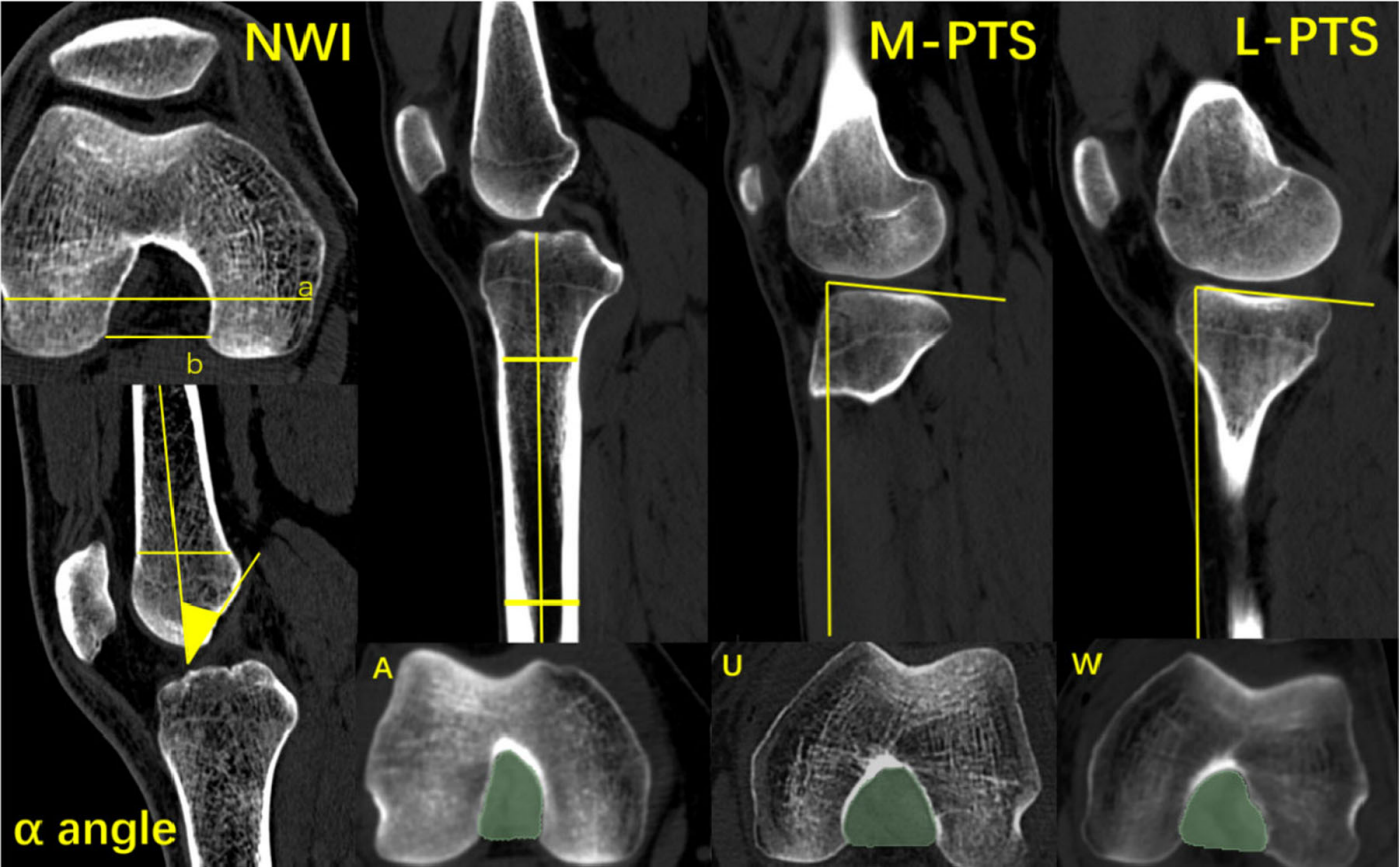
The notch width index (NWI) is defined as follows: line ‘a’ is the length of the medial and lateral femoral condyles, parallel to the posterior edge of the condyles at the plane passing through the popliteal tendon hiatus. b denotes the notch width, which is measured as the line segment at the exit of the intercondylar notch and is also oriented parallel to the posterior edge of the medial and lateral femoral condyles. Finally, the notch width index is calculated as the ratio of b to a (NWI = b/a). The femoral α angle is defined as the angle formed between the long axis of the femur and the Blumensaat line. The long axis of the femur is determined as the straight line connecting the midpoints of the anterior and posterior cortices, measured at a specific distance from the lower edge of the femoral condyles. Posterior tibial slope (PTS): a – The midline is defined as the line connecting the midpoints of the anterior and posterior cortices, measured at 5 cm and 15 cm distal to the tibial plateau along the long axis of the tibia. b – medial posterior tibial slope (M‐PTS) is the angle formed between the vertical line perpendicular to the long axis of the tibia and the tibial plateau, measured at the sagittal level corresponding to the midpoint of the medial tibial plateau. c – lateral posterior tibial slope (L‐PTS) is the angle formed between the vertical line perpendicular to the long axis of the tibia and the tibial plateau, measured at the sagittal level corresponding to the midpoint of the lateral tibial plateau. The morphology of the intercondylar fossa is classified into three types: Type A: The intercondylar fossa exhibits an ‘A’‐shaped configuration. The narrowing of the notch from its entrance to the apex resembles the letter ‘A’, as indicated by the green area. Type U: The intercondylar fossa presents a ‘U’‐shaped morphology. Compared to Type A, it is characterised by a wider apex, resembling an inverted letter ‘U’. Type W: The femoral notch displays a ‘W’‐shaped contour. This morphology is distinguished by the presence of two distinct apices on the notch roof.

Demographic data, including age, sex, height, weight, and body mass index (BMI), were recorded. Clinical parameters based on Computed Tomography (CT) measurement included medial posterior tibial slope (M‐PTS) [2], lateral posterior tibial slope (L‐PTS) [2], notch width index (NWI) [1, 19], femoral‐ɑ‐angle [3], intercondylar notch width [1], femoral condylar width [1], notch morphology (classified as A, U or W) [1] (Figure 1). Radiomic features were extracted from the intercondylar notch region. Through radiomics, the 3D morphology of the intercondylar notch was quantified, yielding the following metrics. This includes Elongation, Least Axis Length, Voxel Volume, Major Axis Length, Sphericity, Maximum 2D Diameter Column, Maximum 2D Diameter Row, Flatness, Maximum 2D Diameter Slice, Maximum 3D Diameter, Mesh Volume, Surface Area, Minor Axis Length and Surface Volume Ratio. The Maximum 2D diameter Column is defined as the largest pairwise Euclidean distance between tumour surface mesh vertices in the row‐slice plane. Other radiomics features can be retrieved through the website (https://pyradiomics.readthedocs.io/en/latest/index.html).

For radiological evaluation, patients were scanned in the supine position with the knee joint fully extended and the lower limb rotated to ensure anterior orientation of the patella. All CT scans were performed in spiral mode. High‐resolution multi‐detector CT was conducted using a Philips CT 256 scanner (Koninklijke Philips N.V.). The scanning parameters were as follows: a 256 × 256 matrix, slice thickness of 1–2 mm, display field of view of 300 mm, pitch of 0.925, rotation time of 0.4 s, and scan time of 1.9 s, with both soft tissue and bone reconstruction kernels applied. The acquired images were downloaded from the PACS and preserved in the original Digital Imaging and Communications in Medicine (DICOM) format. Images reconstructed using the bone kernel were selected for further analysis. All clinical data measurements and radiomic feature extraction were carried out using 3D Slicer (version 5.6.2, https://www.slicer.org/). All imaging measurements were independently performed by two senior orthopaedic specialists.

**Figure 2:**
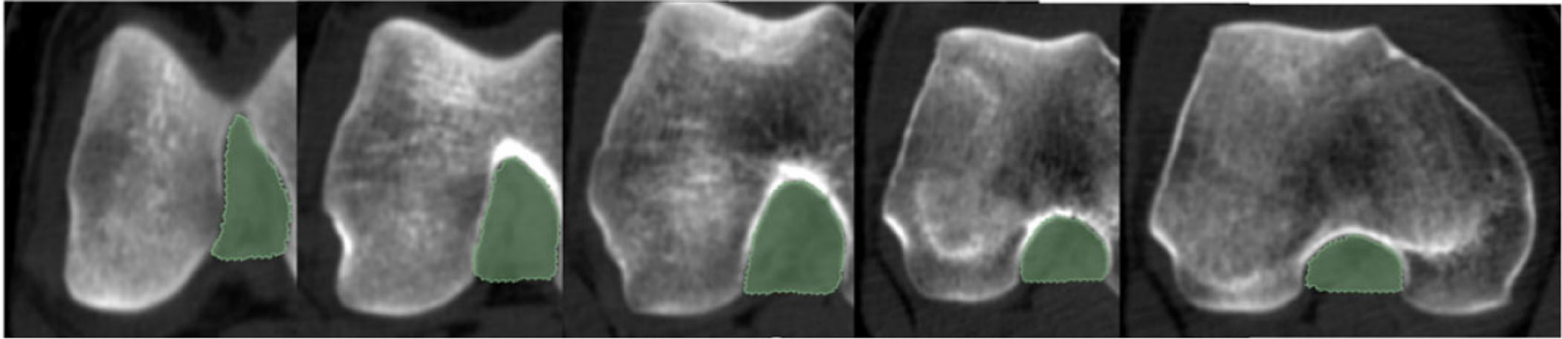
The 3D morphology of the intercondylar fossa was reconstructed and analysed. The delineation extended from the initial continuous plane formed by the bone of the medial and lateral femoral condyles to the plane corresponding to the uppermost point of Blumensaat's line. The posterior boundary was defined as the line connecting the points where the slope of the cortical tangent of the medial and lateral femoral condyles exhibits the most abrupt change. The morphology of the condylar fossa was progressively delineated in a layer‐by‐layer manner.

For radiomics data quantification, all DICOM‐format images were reformatted to standardise the data. The reformatted images had the following characteristics: image dimensions of 256 × 256, slice thickness of 0.5 mm, voxel spacing of 0.5 mm × 0.5 mm × 0.5 mm, and a window width range of 400–1000. Region of interest (ROI) covering the intercondylar fossa was manually delineated on multiplanar reformatted images across the axial, sagittal and coronal planes. Radiomic feature extraction from the ROI was conducted using the PyRadiomics toolbox (version 3.1.0). The extracted parameters included, but were not restricted to, three‐dimensional geometric features such as Elongation and Flatness (Figure 2).

## MACHINE LEARNING PROCESS

**Figure 3:**
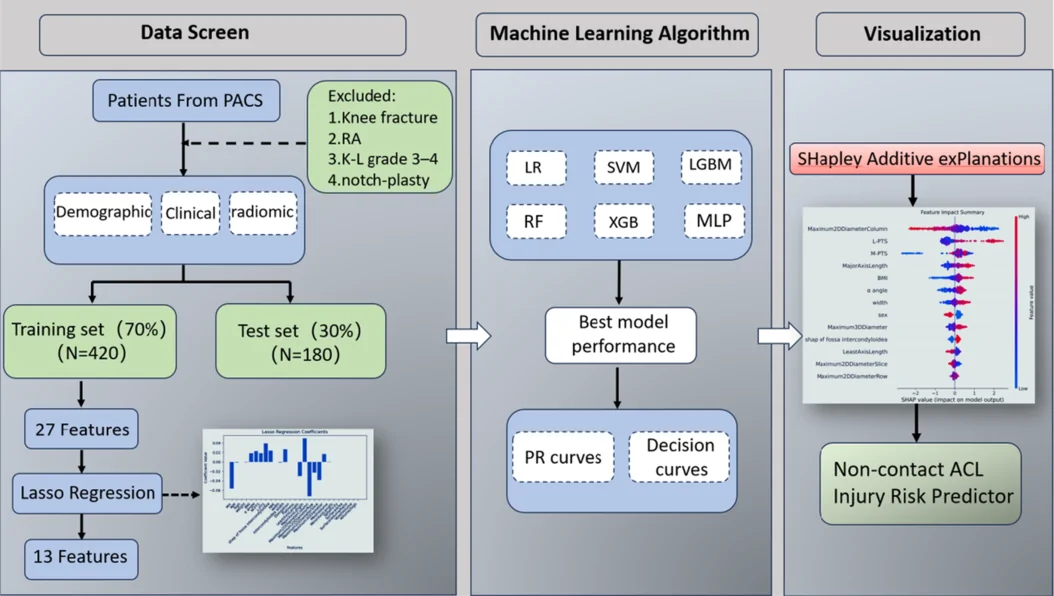
Analysis flow for the development and evaluation of the machine learning models. BMI, body mass index; LGBM, light gradient boosting machine; LR, logistic regression; MLP, multilayer perceptron; MRI, magnetic resonance imaging; PACS, Picture Archiving and Communication System; RA, rheumatoid arthritis; RF, random forest; SVM, support vector machine; XGB, extreme gradient boosting classifier.

Figure 3 presents a schematic illustration of the process used to develop the machine learning algorithms in this study. The procedure included three main stages: data Screen, model development, and model interpretation. A total of 27 features, categorised into demographic, clinical, and radiomic features, were used as input variables. To facilitate model training and evaluation, the dataset was randomly divided into a training set and a test set at a 7:3 ratio. Stratified sampling was employed to ensure a balanced distribution of ACL injury and non‐injury cases in both the training and test sets. Additionally, all variables were standardised before model implementation.

**Figure 4:**
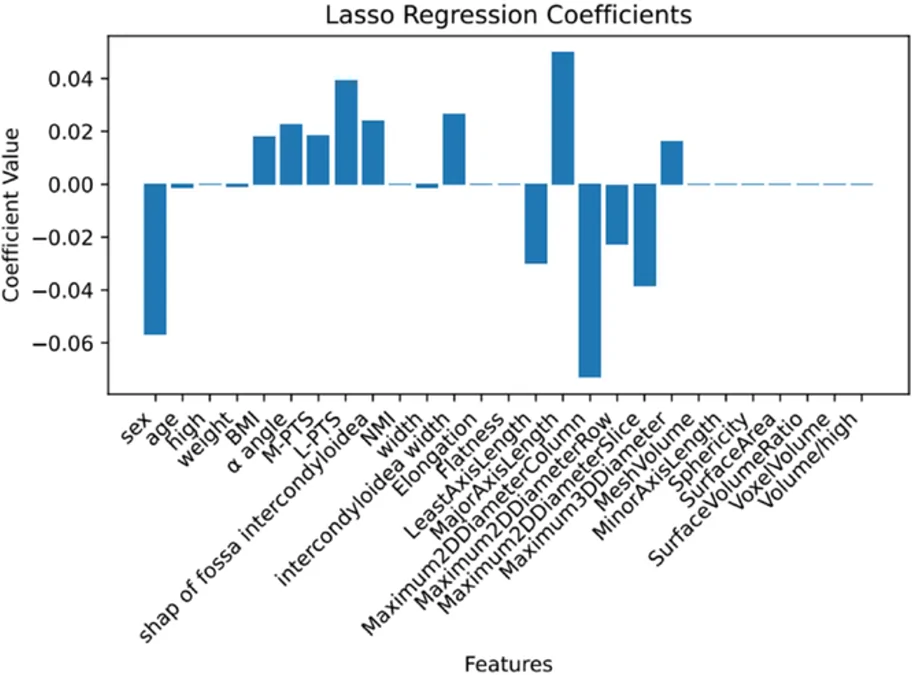
LASSO regression coefficients with feature selection. BMI, body mass index; L‐PTS, Lateral posterior tibial slope; M‐PTS, Medial posterior tibial slope.

Feature selection and data dimensionality reduction were performed using LASSO regression to identify and retain the most relevant features, while eliminating those with coefficient values approaching zero. Following this process, the feature set was reduced to thirteen: gender, BMI, femoral α angle, M‐PTS, L‐PTS, intercondylar fossa morphology, Least Axis Length, Major Axis Length, Maximum 2D Diameter Column, Maximum 2D Diameter Row, intercondylar width, Maximum 2D Diameter Slice and Maximum 3D Diameter (Figure 4). The resulting dimension‐reduced dataset was then used to develop of machine learning models.

A total of six algorithms – logistic regression (LR), support vector machine (SVM), random forest (RF), extreme gradient boosting classifier (XGB), light gradient boosting machine (LGBM) and multi‐layer perceptron (MLP) – were employed to develop predictive machine learning models. The optimised model was evaluated using an independent test set. The Area Under the Precision‐Recall Curve was selected as the primary metric for assessing the model's predictive performance. All model development procedures were implemented using Scikit‐learn (version 1.0.2), XGBoost (version 0.90), LGBM (version 2.2.3) and Python (version 3.7.15).

The Shapley Additive Explanations (SHAP) analysis was employed for model interpretation [21]. First, the individual SHAP values for all participants were calculated. Subsequently, the SHAP values for each feature were ranked by their importance and visualised in a summary plot. Furthermore, the relationship between each predictive feature and the corresponding SHAP values was analysed using a dependency plot.

All statistical analyses were performed using Python (3.7.15), NumPy, and SciPy. For the delineation of ROI and the measurement of clinical characteristics, inter‐observer and intra‐observer measurement reliability was assessed using the intraclass correlation coefficient. G*Power (3.1.9.7) was employed to calculate the required sample size. An a priori power analysis was conducted to determine the minimum number of patients needed in each group. According to the a priori power analysis, at least 249 patients were required in each group (*α* = 0.05, *β* = 0.95). Continuous variables were compared between the two groups using the *t*‐test and Mann–Whitney *U* test, while categorical variables were analysed using the Pearson χ² test or Fisher's exact test. The significance level was set at *p* < 0.05.

## RESULTS

The inter‐observer and intra‐observer agreement for measuring clinical parameters and delineating radiomic ROI was excellent, with mean intraclass correlation coefficients of 0.89 (range, 0.83–0.94) and 0.94 (range, 0.9–0.96), respectively. A total of 600 knees were included in this study, consisting of 400 patients with ACL injuries and 200 patients in the intact control group. No significant differences were observed in demographic characteristics between the two groups with respect to sex or age (Table 1). The Maximum 2D Diameter Column values for the non‐contact ACL injury group and the intact ACL group were 31.12 ± 2.92 mm (95% confidence interval [CI]: 30.83–31.40, *p* < *0.05*) and 32.37 ± 3.07 mm (95% CI: 31.94–32.80, *p* < *0.05*), respectively (Table 2).

**Table 1:** Demographic characteristic.

	Total	ACL intact	ACL injury	*p* value
Sex (female/male)	210/390	98/102	112/288	＞0.05
Age (years)	29.1 ± 7.2	30.1 ± 7.8	28.6 ± 7.2	＞0.05
BMI (kg/m^2^)	23.82 (21.50–26.26)	22.87 (20.03–25.69)	24.10 (22.19–26.78)	＜0.05*

Abbreviation: BMI, body mass index.

* indicates statistical significance

**Table 2:** Radiomic features.

	Total	ACL intact	ACL injury	*p* value
Elongation	0.75 (0.69–0.80)	0.76 (0.70–0.81)	0.74 (0.68–0.80)	＜0.05*
Flatness	0.61 (0.58–0.63)	0.62 (0.59–0.64)	0.60 (0.57–0.63)	＞0.05
Least axis Length (mm)	17.42 ± 1.30	17.59 ± 1.38	17.34 ± 1.25	＜0.05*
Major axis Length (mm)	28.7 ± 2.58	28.37 ± 2.39	28.86 ± 2.65	＜0.05*
Maximum 2D diameter column (mm)	31.54 ± 3.03	32.37 ± 3.07	31.12 ± 2.92	＜0.05*
Maximum 2D diameter row (mm)	35.19 (32.80–37.10)	34.79 (32.80–36.42)	35.40 (32.80–37.48)	＞0.05
Maximum 2D diameter slice (mm)	27.85 ± 2.39	28.38 ± 2.50	27.59 ± 2.29	＜0.05*
Maximum 3D diameter (mm)	37.19 (35.01–39.54)	36.76 (34.85–38.58)	37.52 (35.23–39.78)	＜0.05*
Mesh volume (mm)	6831 ± 1433	6769 ± 1441	6862 ± 1430	＞0.05
Minoraxis length (mm3)	21.41 ± 2.17	21.39 ± 2.16	21.43 ± 2.18	＞0.05
Sphericity	0.68 (0.63–0.71)	0.67 (0.58–0.69)	0.68 (0.65–0.71)	＜0.05*
Surface area	2620 (2306–2903)	2619 (2352–2904)	2587 (2278–2896)	＞0.05
Surface volume ratio	0.37 (0.35–0.41)	0.37 (0.35–0.40)	0.38 (0.36–0.43)	＜0.05*
Voxel volume (mm^3^)	6847 ± 1435	6787 ± 1443	6876 ± 1432	＞0.05

The M‐PTS in the non‐contact ACL injury group and the intact ACL group were 8.86 (95% CI: 7.28–10.19, *p* < 0.05) and 7.64 (95% CI: 5.50–9.14, *p* < 0.05), respectively. The L‐PTS were 10.01 (95% CI: 7.80–11.65, *p* < 0.05) and 8.04 (95% CI: 7.12–9.26, *p* < 0.05), respectively (Table 3).

Abbreviations: Please refer to the Supplement radiomics for further details.

* indicates statistical significance

**Table 3:** Clinical characteristics.

	Total	ACL intact	ACL injury	*p* value
NMI	0.27 ± 3.81	0.27 ± 0.03	0.27 ± 0.03	＞0.05
Femoral ɑ angle (°)	38.26 ± 3.20	37.28 ± 3.06	38.75 ± 3.16	＜0.05*
M‐PTS (°)	8.47 (6.77–9.87)	7.64 (5.50–9.14)	8.86 (7.28–10.19)	＜0.05*
L‐PTS (°)	8.99 (7.49–10.95)	8.04 (7.12–9.26)	10.01 (7.80–11.65)	＜0.05*
Width (mm)	70.24 (64.32–72.79)	67.92 (63.47–72.36)	70.67 (65.60–73.04)	＜0.05*
notch width (mm)	18.76 ± 3.05	18.61 ± 3.08	18.84 ± 3.03	＞0.05

Abbreviations: NWI, notch width index; PTS, posterior tibial slope.

* indicates statistical significance

**Figure 5:**
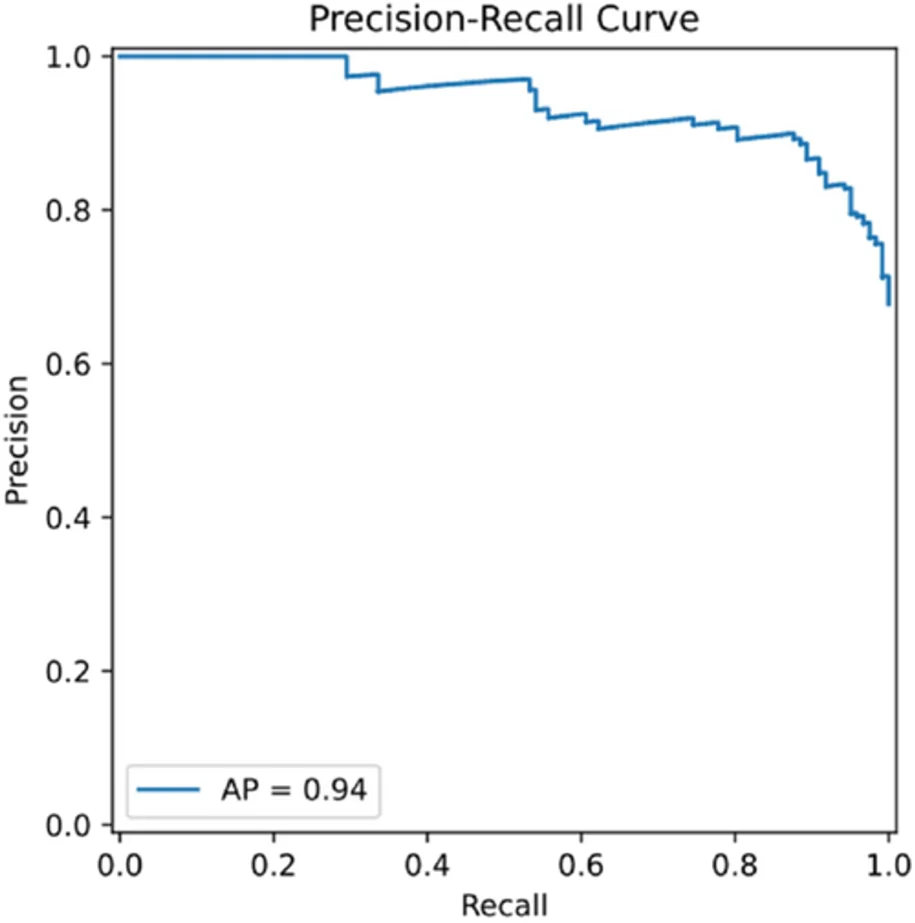
The precision–recall curve. AP, area under the precision–recall curve = 0.94.

The predictive performance of machine learning models: Through comparison of the RF(AUCOR = 0.82), SVM(AUCOR = 0.83), LGBM(AUCOR = 0.87), LR(AUCOR = 0.85), XGB(AUCOR = 0.89), and MLP(AUCOR = 0.84) models. In this study, the XGBoost model was identified as the optimal model in this study. The key parameter settings were as follows: column sampling ratio per tree was 0.8, learning rate was 0.05, maximum depth was 7, minimum node weight was 2, number of trees was 100, and subsampling ratio was 0.7. This model achieved an accuracy of 94% in predicting non‐contact ACL injuries (Figure 5), demonstrating its effectiveness in identifying individuals at risk for such injuries.

**Figure 6:**
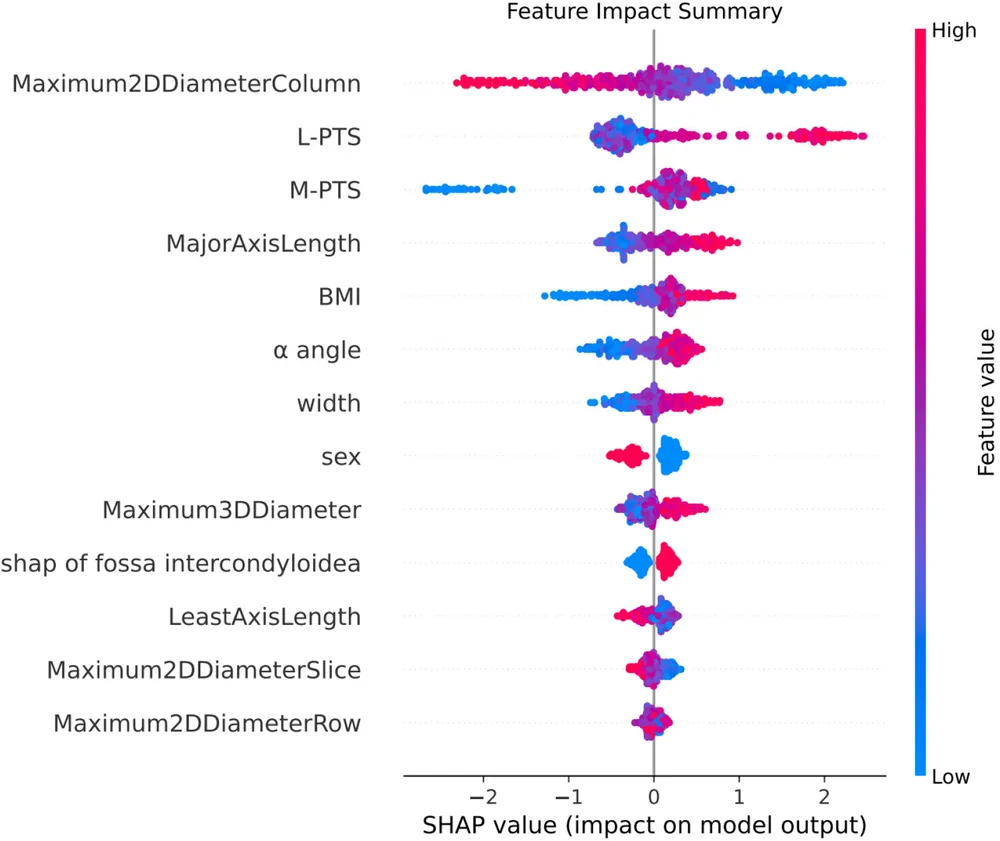
Shapley additive explanation (SHAP) summary plot. All features are ordered by SHAP importance. Each dot represents a sample coloured by its feature value. BMI, body mass index; L‐PTS, lateral posterior tibial slope; M‐PTS, medial posterior tibial slope.

**Figure 7:**
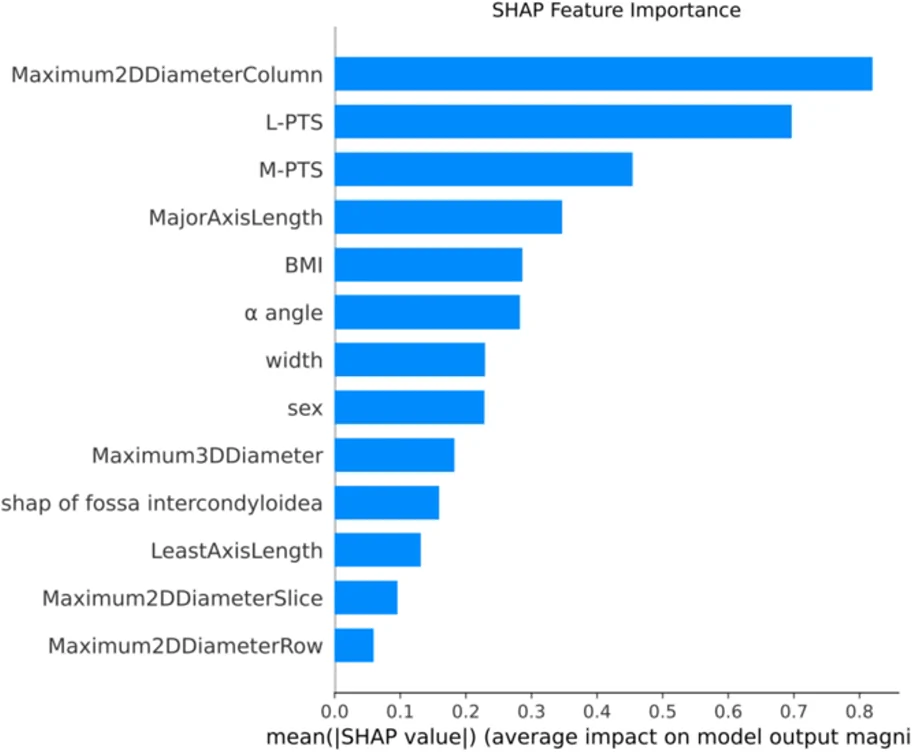
Shapley additive explanation (SHAP) feature importance. BMI, body mass index; L‐PTS, Lateral posterior tibial slope; M‐PTS, Medial posterior tibial slope.

**Figure 8:**
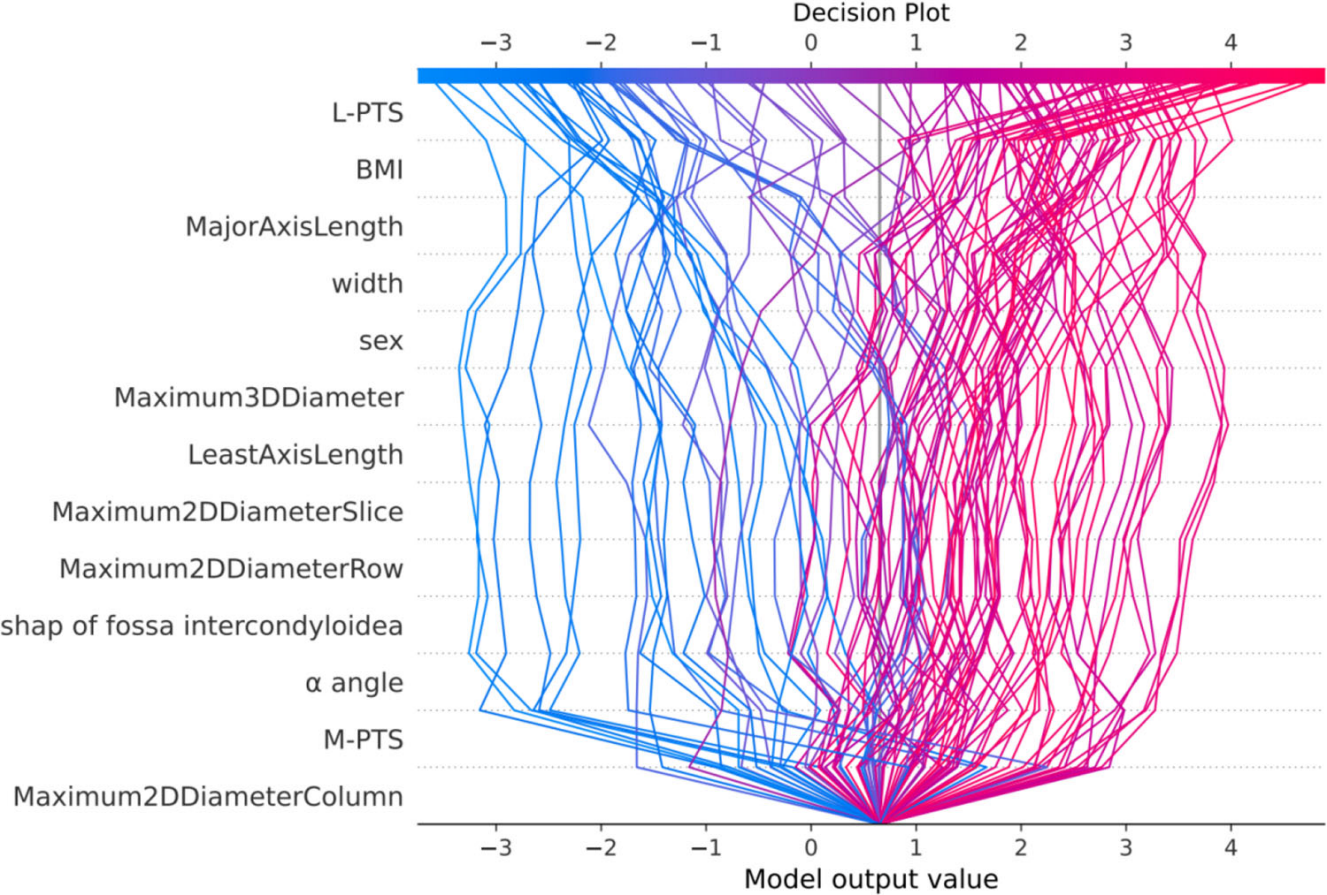
Shapley additive explanation (SHAP) decision plot. All features are ordered by SHAP importance. Each lane represents a sample coloured by its feature value. BMI, body mass index; L‐PTS, lateral posterior tibial slope; M‐PTS, medial posterior tibial slope.

Key Predictors of ACL Injury: Feature importance analysis of the XGBoost model (Figures 6, 7) was conducted using SHAP. The Maximum 2D Diameter Column was identified as the most important predictor of non‐contact ACL injury, followed by the lateral posterior tibial slope, medial posterior tibial slope, and Major Axis Length. Figure 8 illustrates the detailed decision‐making process of the XGBoost model.

## DISCUSSION

The most significant finding of this study is that the Maximum 2D Diameter Column of the intercondylar fossa was identified as the most critical factor influencing the risk of non‐contact ACL injury through the XGB model. Furthermore, the machine learning model demonstrated strong predictive performance for non‐contact ACL injuries. This study can use preoperative CT to determine whether patients with non‐contact ACL injuries have associated bony risks related to the intercondylar notch, providing personalised treatment plans for the patients. If necessary, a notchplasty can be performed to address a narrowed intercondylar notch.

Radiomics converts images into mineable data and then analyses these data to support decision‐making [14]. Radiomics offers a vast and potentially limitless range of imaging biomarkers that may significantly contribute to cancer detection, diagnosis, prognostic assessment, prediction of treatment response, and ongoing disease monitoring [22, 31]. Although this method has been applied in the diagnosis of meniscus and ACL injuries, there is limited research on the radiomics of the intercondylar fossa.

In terms of predictive performance, XGB demonstrated superior accuracy. It has been reported that generating multiple trees to reduce prediction error yields satisfactory results [24]. An increasing number of researchers have focused on identifying risk factors for non‐contact ACL injuries. Studies have shown that, in addition to knee osteoarticular factors, several other variables influence the occurrence of such injuries, including joint laxity, reduced lower limb muscle strength, and poor knee joint stability during landing. Therefore, models based solely on the analysis of knee geometric characteristics may be limited in their ability to achieve high predictive accuracy [20, 27, 36].

The 2D assessment of intercondylar notch morphology, such as the NWI, has been widely utilised in previous studies [12, 17, 19]. Iriuchishima et al. reported that a smaller NWI is correlated with a higher incidence of ACL injury [17]. However, in this study, no significant difference in NWI was observed between the ACL injury group and the control group. This discrepancy may be attributed to the measurement method of NWI, which is based on the level of the popliteal tendon at the femoral medial and lateral condyles, potentially leading to variations in results. It has also been documented that the shape of the intercondylar notch outlet is associated with the risk of ACL injury [32]. A larger femoral α angle has been linked to an increased likelihood of ACL injury [1, 28], although the underlying mechanism remains to be fully elucidated. Fernandez‐Jaen et al. suggested that a larger femoral α angle causes the ACL to impinge on the anterior intercondylar notch during knee extension, and repetitive extension movements may contribute to ACL injury [13]. In this study, the femoral α angle was significantly greater in the non‐contact ACL injury group compared to the ACL‐intact group (*P* < 0.05), further supporting its role as a potential risk factor for ACL injury. Other studies have demonstrated that an increased PTS is a significant risk factor for ACL injury. Multiple independent studies have consistently reported statistically significant differences in PTS between ACL‐injured and ACL‐intact group [5, 37]. Brandon M.L. et al. utilised lateral X‐ray films and aligned measurements with the tibial long axis to show that patients with ACL injuries exhibited a greater PTS [9]. Hohmann E et al., based on knee MRI data, found that both M‐PTS and L‐PTS were potential risk factors for ACL injury in both genders [16]. However, previous studies have suggested that only L‐PTS is a risk factor for ACL injury in PTS [11, 38]. Brandon M.L. et al. suggested that PTS significantly influences anterior‐posterior joint stability, in situ forces on the ACL graft, and anterior shear forces [9]. Webb et al and Wen et al used MRI and CT imaging and found high variability in the consistency of M‐PTS and L‐PTS measurement [34, 35]. A larger PTS increases ACL tension during movement, thereby increasing the likelihood of injury. In this study, CT‐based measurements revealed that both medial and lateral PTS were significantly greater in the ACL injury group compared with the ACL‐intact group (*P* < 0.05). However, these findings differed significantly from those of Wen et al., which may be attributed to variations in the method of measuring the tibial long axis. A type A narrow femoral notch has been identified as an important risk factor for ACL injury [1, 8, 33], and these studies collectively emphasise the strong association between knee joint bone geometry and non‐contact ACL injury. Nevertheless, a notable limitation is the lack of a comprehensive analysis of these geometric characteristics has not yet been conducted.

Based on this model, the maximum 2D diameter column is proposed as a novel two‐dimensional morphological index for assessing the intercondylar notch of the femur. The Maximum 2D Diameter Column represents the largest two‐dimensional diameter between any two points within the ROI in the sagittal plane. This parameter directly quantifies the size of the intercondylar notch in the sagittal plane. A smaller intercondylar notch has been associated with an increased risk of non‐contact ACL injury. Bouras T et al. reported that a narrow intercondylar notch is a risk factor for non‐contact ACL injury [8]. In contrast, this study applies radiomics to reduce dimensionality and objectively quantify the morphology of the intercondylar notch, revealing that patients with non‐contact ACL injuries tend to have a smaller Maximum 2D Diameter Column.

This study has several limitations. First, the dataset was collected from a single institution, which could limit the generalisability of the findings. Second, the sample size was relatively small. To validate the generalisability of our machine learning model, its performance should be evaluated using independent datasets in accordance with established guideline [15]. Further multi‐centre, large‐scale studies are warranted. Third, all anatomic features that were manually determined were based on CT images, which may differ from more conventional radiographs. For instance, posterior tibial slope is most often measured using full length tibia or lateral knee films. Fourth, a smaller intercondylar notch is associated with a smaller ACL, which in turn may increase the risk of non‐contact ACL injury. Due to measurement limitations, the volume of the ACL was not considered in this study. Fifth, this was a retrospective study, and the data from patients with non‐contact ACL injury were collected preoperatively, rather than through prospective follow‐up. Therefore, caution should be exercised when interpreting the feature importance results. Future biomechanical studies are necessary to investigate the clinical relevance of these findings and to further validate and expand upon the conclusions of this study.

## CONCLUSION

The machine learning model developed in this study demonstrated excellent predictive performance for non‐contact ACL injuries. The Maximum 2D Diameter Column was the most important predictor, followed by the lateral and medial posterior tibial slope.

## AUTHOR CONTRIBUTIONS

Ke Xiao was responsible for the organisation and coordination of this study. Ke Xiao, Song Wu, Jinshen He, Chi Liang, Yangbo Cao and Jiewen Luo participated in study design. Ke Xiao and Damaris Topola Boko performed the statistical analysis. Ke Xiao and Damaris Topola Boko collected and interpreted the data. Jinshen He conceived of the study, participated in coordination and helped to draft the manuscript. Bosomtwe Kwabena Richmond, Yunheng Yao, Benjamin Rothrauff and Jinshen He revised the manuscript. All authors read and approved the final manuscript.

## CONFLICT OF INTEREST STATEMENT

The authors declare no conflicts of interest.

## ETHICS STATEMENT

Institutional Review Board approval was obtained before performing the study (Approval No. 2025‐S471).

## Supporting information

Supplement table.

Supplement radiomic.

## Data Availability

Data are available on reasonable request from the authors.
