# Supplementary material for: The maximum 2D diameter column of the notch as the most important bone risk indicator compared with the posterior tibial slopes for ACL injury based on computed tomography: Analysis using machine learning approach
Source: J Exp Orthop. 2026 Jan 11;13(1):e70630. doi: 10.1002/jeo2.70630 (PMC12793037; doi:10.1002/jeo2.70630)
Supplement: Supplementary file 1 — Supplement table. [file JEO2-13-e70630-s001.docx]

**Supplement table1: reliability of ROI measurement**

|  | **ICC(95%CI)** |
| --- | --- |
| Inter-observer | 0.89 (range, 0.83–0.94) |
| Intra-observer | 0.94 (range, 0.90–0.96) |

The consistency inter- and intra- observer is robust and well-established.
