# Supplementary material for: The maximum 2D diameter column of the notch as the most important bone risk indicator compared with the posterior tibial slopes for ACL injury based on computed tomography: Analysis using machine learning approach
Source: J Exp Orthop. 2026 Jan 11;13(1):e70630. doi: 10.1002/jeo2.70630 (PMC12793037; doi:10.1002/jeo2.70630)
Supplement: Supplementary file 2 — Supplement radiomic. [file JEO2-13-e70630-s002.docx]

**The meaning of Radiomic features**

Elongation: Elongation shows the relationship between the two largest principal components in the ROI shape.

Least Axis Length: This feature yield the smallest axis length of the ROI-enclosing ellipsoid.

Voxel Volume: The volume of the ROI V voxel is approximated by multiplying the number of voxels in the ROI by the volume of a single voxel

Major Axis Length: This feature yield the largest axis length of the ROI enclosing ellipsoid.

Sphericity: Sphericity is a measure of the roundness of the shape of the tumor region relative to a sphere.

Maximum 2D Diameter Column: Maximum 2D diameter Column is defined as the largest pairwise Euclidean distance between tumor surface mesh vertices in the row-slice (usually the coronal) plane.

Maximum 2D Diameter Row: Maximum 2D diameter Row is defined as the largest pairwise Euclidean distance between tumor surface mesh vertices in the column-slice (usually the sagittal) plane.

Flatness: Flatness shows the relationship between the largest and smallest principal components in the ROI shape.

Maximum 2D Diameter Slice: Maximum 2D diameter Slice is defined as the largest pairwise Euclidean distance between tumor surface mesh vertices in the row-column (generally the axial) plane.

Maximum 3D Diameter: Maximum 3D diameter is defined as the largest pairwise Euclidean distance between tumor surface mesh vertices.

Mesh Volume: The volume of the ROI V is calculated from the triangle mesh of the ROI.

Surface Area: The surface area of ROI

Minor Axis Length: This feature yield the second-largest axis length of the ROI enclosing ellipsoid.

Surface Volume Ratio: The ratio of surface area to volume.
